# Au@Ag Dendritic Nanoforests for Surface-Enhanced Raman Scattering Sensing

**DOI:** 10.3390/nano11071736

**Published:** 2021-06-30

**Authors:** Hung Ji Huang, Ming-Hua Shiao, Yang-Wei Lin, Bei-Ju Lin, James Su, Yung-Sheng Lin, Han-Wei Chang

**Affiliations:** 1 Taiwan Instrument Research Institute, National Applied Research Laboratories, Hsinchu 300092, Taiwan; hjhuang@narlabs.org.tw (H.J.H.); mhshiao@tiri.narl.org.tw (M.-H.S.); sujames@tiri.narl.org.tw (J.S.); 2 Department of Chemistry, National Changhua University of Education, Changhua 500207, Taiwan; linywjerry@cc.ncue.edu.tw; 3 Department of Chemical Engineering, National United University, Miaoli 360001, Taiwan; u0714108@gm.nuu.edu.tw; 4 Ph.D. Program in Materials and Chemical Engineering, National United University, Miaoli 360001, Taiwan; 5 Institute of Food Safety and Health Risk Assessment, National Yang Ming Chiao Tung University, Taipei 112304, Taiwan

**Keywords:** Au@Ag dendritic nanoforests, fluoride-assisted Galvanic replacement reaction, surface-enhanced Raman scattering, surface plasmons

## Abstract

The effects of Au cores in Ag shells in enhancing surface-enhanced Raman scattering (SERS) were evaluated with samples of various Au/Ag ratios. High-density Ag shell/Au core dendritic nanoforests (Au@Ag-DNFs) on silicon (Au@Ag-DNFs/Si) were synthesized using the fluoride-assisted Galvanic replacement reaction method. The synthesized Au@Ag-DNFs/Si samples were characterized using scanning electron microscopy, energy-dispersive X-ray spectroscopy, reflection spectroscopy, X-ray diffraction, and Raman spectroscopy. The ultraviolet-visible extinction spectrum exhibited increased extinction induced by the addition of Ag when creating the metal DNFs layer. The pure Ag DNFs exhibited high optical extinction of visible light, but low SERS response compared with Au@Ag DNFs. The Au core (with high refractive index real part) in Au@Ag DNFs maintained a long-leaf structure that focused the illumination light, resulting in the apparent SERS enhancement of the Ag coverage.

## 1. Introduction

SERS measurements typically utilize the induced surface plasmons on a metal surface to chemically or physically enhance the intensity of Raman scattering [1,2,3,4,5,6,7,8,9,10]. SERS is an ultrasensitive vibrational spectroscopic technique used to detect molecules near the surface of plasmonic nanostructures. SERS can be utilized in chemical, material, and life sciences [4,5]. Many methods have been developed to yield higher SERS responses, including the use of rough metal surfaces [11], various sizes of metal nanoparticles (NPs) [6,12,13], composited NPs [3,6,14], Ag film over a nanosphere structure [8], a hyperbolic metamaterial (HMM) structure [7], and Ag dendritic nanoforests [10]. Using SERS of individual Ag colloidal NPs can achieve optical detection and spectroscopy of single molecules and NPs at room temperature [2]. Flexible SERS sensors were achieved through the fabrication of Ag–Au core-shell NPs based on chemical reduction and Galvanic replacement processes on a polyimide (PI) substrate [9].

There are already a lot of research works regarding two-dimensional SERS enhancement substrates, e.g., fixed monolayer nanoparticles, patterned metallic nanostructures. These single-layer SERS sensors work very well with a high enhancement ratio. Clearly developed theoretical electromagnetic modeling also demonstrated ultra-high enhancement on specific demanded spectrum areas. However, these two-dimensional SERS enhancement substrates typically have only monolayer surface area and can adsorb little target molecules. The two-dimensional structure is also not easy to capture target molecules in microfluidics. Metal or dielectric DNFs structure [10,15,16,17,18,19,20,21,22,23,24,25,26,27,28,29,30] has complex three-dimensional cross-linking networks to fix many target molecules. Chan et al. presented that the Ag dendrites formed and protected by a 2 nm SiO_2_ film on a Si surface [17] demonstrated a high sensitivity of 10^−8^ M R6G Raman response. The proposed Ag-based SERS sensor showed excellent chemical stability even store in the air for as many as 50 days. Therefore, metal DNFs structures are promising SERS substrates to have high measurement sensitivity. The metal DNFs typically have vastly increased growth height up to several μm and should have much different surface morphology as comparing to the nanoparticles of feature size typically smaller than 100 nm. Advanced studies are demanded to have clear understanding on electromagnetic enhancement among various factors for nanometer sized nanospheres and μm height DNFs.

Bimetallic Au–Ag NPs offer the advantage of enhanced SERS signal broadband extinction of light [6,31]. Au–Ag NPs also have benefits in terms of chemical stability for long-term use. The flexible SERS sensor consisted of Ag and Au core-shell NPs on a PI substrate exhibited superior efficiency and durability after storage for 30 days and even after 500 cycles of mechanical stimuli (bending or torsion) [9]. Ag-based hybrid nanoprobes have shown their tremendous potential for SERS imaging of precise biological detection and mediated phototherapy [32]. High-performance SERS two-dimensional nanodot array was designed through liquid–liquid interfacial self-assembly of the core-shell nanoparticles (Au@Ag NPs) and exploited to assess dual-fungicides in pear, apple, and orange juices [33]. The 2D Au@Ag nanodot array delivered good uniformity and reproducibility with the substrate-to-substrate relative standard deviation values of 10.51% [33]. Dual-channel colorimetric and SERS strategy was developed for detection of Cu^2+^ utilizing Ag–Au core-satellite nanostructures [34]. Gold-coated silver (Ag–Au) and silver-coated gold (Au–Ag) composite nanoparticles are prepared by a seeding growth method to study their SERS-active properties [35]. Among the metals used, silver (Ag) has been demonstrated to have a more significant enhancement factor (EF) than gold (Au) due to the absence of interband absorption [36,37,38,39]. The Au–Ag film shows a much stronger SERS signal for R6G than those from pure Au, Ag, and Ag–Au films, indicating that the Au–Ag film is more potent than pure Ag and Au film as SERS-active substrates [35]. Ag−Au hybrid nanosponges (NSs) fabricated through the cyclic electroless deposition of Ag into the porous Au NSs presented higher sensitivity in surface-enhanced Raman spectroscopy (SERS) detection of butter yellow than the Au NSs due to the presence of Ag and the porous structure [40]. Cost-effective co-sputter deposited Au–Ag film on pre-patterned Si surface (Au_0.5_Ag_0.5_@P-Si) shown ∼28 times enhanced SERS signal as compared to that of pure Au@P-Si and ∼1.5 times to that of pure Ag@P-Si [41]. The alloy Au_0.5_Ag_0.5_@P-Si exhibited high SERS sensitivity, homogeneity, reproducibility, and chemical stability far beyond those of the individual elements.

Au and Ag are metals of almost the same cell dimension and crystal structure. Ag has a higher plasmonic response but lower chemical stability comparing to Au. However, the previous reference works demonstrated that the Ag–Au composited nanoparticles could have higher SERS performance than pure Au or Ag nanoparticles. The Ag to Au ratio of Ag–Au NPs affects the SERS response. Rivas et al. reported that increased coverage of a Au core with a Ag shell induces an increase in the enhancement factor of SERS response compared with pure Au [3]. Freeman et al. suggested that the SERS behavior of aggregated colloids is highly dependent on the Au/Ag ratio of Au–Ag NPs [1]. Tiny amounts of Ag can increase SERS intensity, but the additional increases of Ag lead to complete signal loss. Pham et al. developed a susceptible SERS sensor based on Ag-shell-covered Au seeds on SiO_2_ NPs [6]. They discovered that the growth of the Ag shell on the surface of the Au NP seeds and the formation of narrow gaps between two Ag NPs on the surface of the probes resulted in increased SERS sensitivity. The NPs exhibited strong Raman signals, which originated from a highly enhanced E-field at the gaps. The Au–Ag composited DNFs have a complex optical response under external light illumination. Therefore, experimental evaluations were motivated to conduct with the SERS sensors of various Ag to Au ratio metal DNFs. This research provides scientific information for developing thick three-dimensional structured SERS sensor layers even though the synthesized Ag base material typically has poor chemical stability.

Results for the SERS enhancement factor [42], $F\left(\lambda \right)$, which are related to various wavelengths of light, can be calculated using the following equation:

(1) $$F\left(\lambda \right)={\left(\frac{E_{loc}}{E_{0}}\right)}_{\lambda_{0}}^{2}{\left(\frac{E_{loc}}{E_{0}}\right)}_{\lambda }^{2}$$

$\lambda_{0}$ is the incident laser wavelength for inducing SERS, and λ represents the wavelength of the SERS response. $E_{0}$ and $E_{loc}$ are the electric field of incident light and the SERS response on the metal surface, respectively. The broadband extinction from the broadband light focus plasmonic effect represents greatly enlarged $E_{loc}/E_{0}$ for incident light ($\lambda_{0}$) and induced SERS light (λ) on the metal surface of NPs or nanostructures deposited with target molecules. Numerous nanogaps between Au–Ag NPs can strengthen and increase the reliability of SERS probes for the sensitive detection of chemicals [6]. Therefore, developing nanometal structures of more particles, gaps, wires, rods, and networks can be promising approaches for improving SERS response [7,8,9,10]. Metal dendritic nanoforests (DNFs) have exhibited a superior plasmonic and SERS response in studies [10,16].

The details of metal DNFs are challenging to be measured even by TEM. The feature sizes of the three-dimensional structures are too large that the electrons can’t penetrate. Therefore, it is difficult to quantitate the EM response of the dendritic nanoforest surface, just like spherical nanoparticles. However, the metal DNFs has benefits that good for SERS measurement. Chan et al. suggested a theoretical study for modeling the metal DNFs in 2013 [17]. In combination with various experimental measurements and theoretical modeling [17] with the calculation of SERS enhancement factor [14], advanced studies can further improve the understanding of metal DNFs sensors.

In this study, we investigated the effects of Ag shells covered Au core DNFs with various Au/Ag ratios. Ag-coated Au DNFs on silicon (Au@Ag-DNFs/Si) were synthesized using the fluoride-assisted Galvanic replacement reaction (FAGRR) method. We considered the strong plasmonic effects of DNFs [10,16,18,19,20] for our SERS analysis.

## 2. Materials and Methods

### 2.1. Preparation of Au@Ag-DNFs/Si Substrate

**Figure 1:**
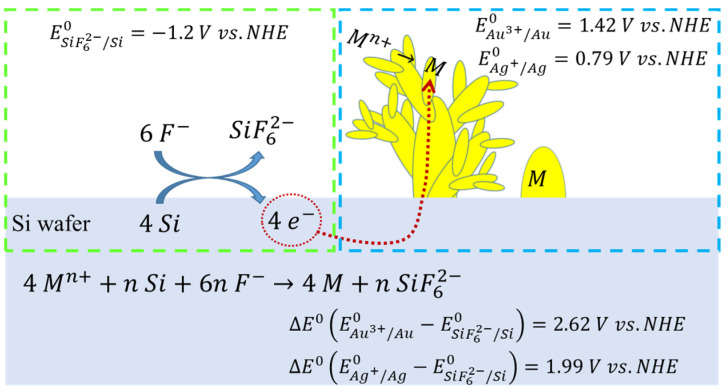
Schematic of the Au@Ag-DNFs/Si Galvanic replacement reaction.

The Au@Ag-DNFs/Si substrate was synthesized using the FAGRR method (Figure 1). The F^−^ in solution reacts with the Si atom, producing SiF_6_^2−^ and releasing four e^−^. The numerous generated e^−^ then flow to suitable positions on the surface of Si, Au, or Ag, and Au^3+^ and Ag^+^ are consequently reduced to Au and Ag, respectively. Thus, the growth of Au and Ag NPs depends on the crystal structure of Au, Ag, e^−^ conductivity in the Ag and the Si substrate, the diffusion of Au^3+^ and Ag^+^ in solution, and the reduction process. Generally, the complex synthesis process produces dendritic forest-like structures [10,16,18,19,20]. In this study, Au was synthesized before Ag.

**Figure 2:**
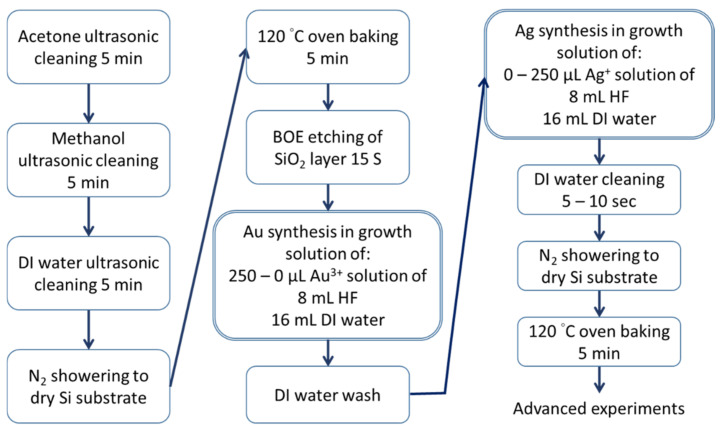
Process of the Galvanic replacement reaction in Au@Ag-DNFs/Si synthesis.

In this study, the synthesis of Au@Ag-DNFs/Si, as illustrated in Figure 2, began with cleaning a 3 × 3 cm^2^ n-type silicon substrate through ultrasonic washing with acetone, methanol, and deionized water, consecutively, for 5 min. The substrate was then dried using an N_2_ spray for 5 min and baked in an oven at 120 °C in a covered glass Petri dish for 5 min. The native oxide layer on the Si substrate was removed by applying a buffered oxide etchant (BOE) solution for 15 s. Hydrofluoric acid (HF) etching can increase the roughness of the Si substrate and the adhesion of the synthesized Au and Ag trees. Si substrates were then treated in a mixture containing 24 mL of reactant solution (8 mL of buffered oxide etchant solution with 11.4% NH_4_F and 2.3% HF, 16 mL of DI water, and various volumes of HAuCl_4_ or AgNO_3_ in a Teflon container measuring 5.5 cm in inner diameter and 4.8 cm in depth). A quantity of 250, 190, 125, 60, and 0 μL of 1 M HAuCl_4_ was added for the Au DNF growth step for sample (A)–(E), wherein; 0, 60, 125, 190, and 250 μL of 1 M AgNO_3_ was added to produce Ag-covered NPs or for the Ag DNF growth step for sample (A)–(E), respectively. The volume of 1 M Au and Ag solutions was fixed at 250 μL for samples with a Au/Ag ratio of 250/0, 190/60, 125/125, 60/190, or 0/250. The synthesized Au@Ag-DNFs were washed three times using deionized water, and the samples were dried using the N_2_ spray and then incubated at 120 °C for 5 min to obtain the Au@Ag-DNFs/Si substrate.

The Au@Ag-DNFs SERS sensor with Ag shell can sulfide fast. The oxidized surface also may result in variation of the sensors’ SERS response. Therefore, we used the Au@Ag-DNFs SERS sensor as quickly as possible when they were fabricated.

### 2.2. Characterization

The material properties of the synthesized Au@Ag-DNFs/Si were characterized using cold-field emission scanning electron microscopy (SEM, SU-8010, Hitachi, Tokyo, Japan), energy-dispersive X-ray spectroscopy (EDX, SU-8010, Hitachi, Tokyo, Japan), and X-ray diffraction (XRD, D8 Discover, Bruker, Billerica, MA, USA). An ultraviolet (UV)-visible reflection spectrophotometer (UV-3101PC, Shimadzu, Kyoto, Japan) with a spherical light integrator was used to measure the reflection spectra of the samples. High-performance Brunauer–Emmett–Teller (BET) surface area and pore size analyzer (Micro 100C, 3P Instruments GmbH & Co. KG, Odelzhausen, Germany) [43] was also utilized to measure the multilayer adsorption on the external surface area of various Ag-DNFs/Si samples.

### 2.3. SERS Analysis

The SERS measurements of the rhodamine 6G (R6G) SERS and 4- mercaptobenzoic acid (4-MBA) were conducted using Raman spectroscopy (UniDRON, UniNanoTech, Korea). The wavelength of incident light is 532 nm with a power of 0.1 mW focusing in a 700 nm spot-diameter while Raman scattering signal accumulation time of 0.5 s. Before the Raman scattering spectrum measurements, the samples were dipped in 10 mL of 10^−6^ M R6G or 4-MBA solution for one day in a glass Petri dish, respectively. After dried through incubation at 37 °C for 24 h, Raman scattering signals were collected using a 100× objective lens (N.A. 0.90) and then detected using a spectrometer. To estimate Raman enhancement factor (EF), typical Raman spectra of R6G and 4-MBA were received by dipping 10 mL of 10^−1^ M R6G or 4-MBA solution on Si wafers for one day, respectively. After dried at 37 °C for 24 h, SERS measurements were performed as described above.

## 3. Results

**Figure 3:**
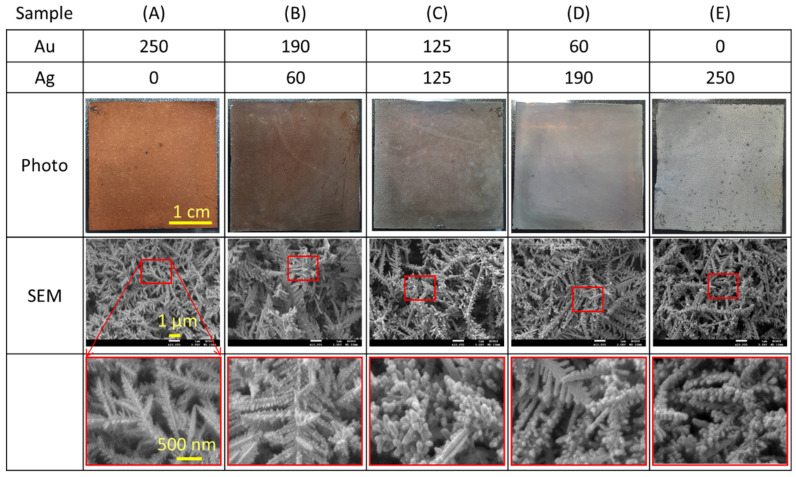
Top-view photo and SEM images of the Au@Ag-DNFs/Si with various Au/Ag ratios (**A**–**E**). The photo and SEM images presented the synthesized Au@Ag-DNFs on the Si substrate with Au/Ag ratios of (**A**) 250/0, (**B**) 190/60, (**C**) 125/125, (**D**) 60/190, and (**E**) 0/250. The bottom pictures are the zoom-in of the red-square marked area.

The photo and SEM images in Figure 3 the synthesized Au@Ag-DNFs on the Si substrate with Au/Ag ratios of (A) 250/0, (B) 190/60, (C) 125/125, (D) 60/190, and (E) 0/250. The Au-rich sample exhibited a deep red color that decreased and transitioned to a bright white color when the proportion of Ag decreased. Because H_2_ gas generated during the Galvanic replacement reaction may significantly influence the morphology of Ag DNFs [10,44], some black holes of small Ag DNFs were evident in the sample (E) with an Au–Ag ratio of 0/250. The SEM pictures revealed that various Au/Ag ratios led to similarly synthesized DNFs. However, the zoomed-in picture of SEM data showed a difference in the microstructure. The Au-rich samples had sharp leaves and branches, whereas the Ag-rich samples tended to exhibit round leaves aggregated on the metal branches.

**Figure 4:**
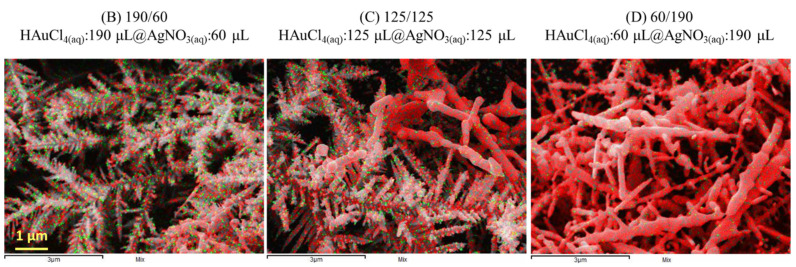
EDX mapping of samples (B–D) in Figure 3. Green indicates Au, and red indicates Ag.

EDX results revealed only Au and Ag signals of samples Figure 4 B–D. Au and Ag have the same crystal structure lattice constant. Therefore, Ag can be densely deposited on Au DNF cores with smooth surface coverage. The pseudo-colored EDX mapping images, with green and red indicating Au and Ag, exhibited a well-synthesized Au core and showed that the Ag shell DNF structure provided stabilized coverage.

**Figure 5:**
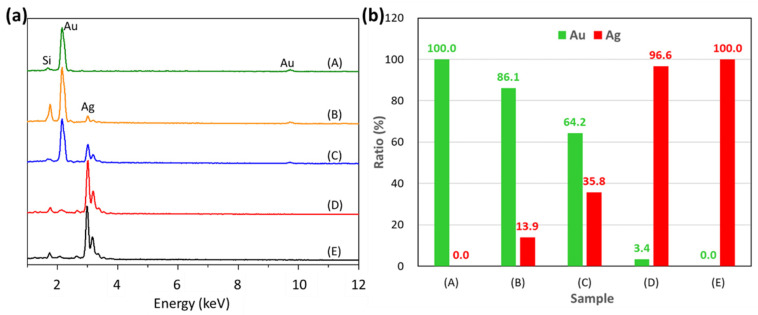
(**a**) EDX data of the various synthesized Au@Ag-DNFs/Si samples. (**b**) Green and red columns denote the respective signals of the Au and Ag of the EDX peak.

As Figure 5a indicates, the Au@Ag-DNFs/Si with various Au/Ag ratios could be estimated using the EDX measurement data. The peaks of Au and Ag were 2.16 keV and 3.0 keV, respectively. The Au (Ag) peaks intensity of samples (A–E) (see Figure 3) decreased (increased) with the Au/Ag ratio decrease, respectively. As illustrated in Figure 5b, the Si and O signals were small and filtered out to estimate the Au and Ag signal ratio. Ag+ ratio peaks increased as the proportion of Ag^+^ increased during synthesis in all samples except for the sample (E). Au was deposited before Ag. The deposited Ag presented to growth only on specific sites of deposited Au. For sample (C), which had a 125/125 Au/Ag ratio, the same concentration during synthesis presented a stronger Au signal despite Au crystals being covered by Ag crystals.

**Figure A1:**
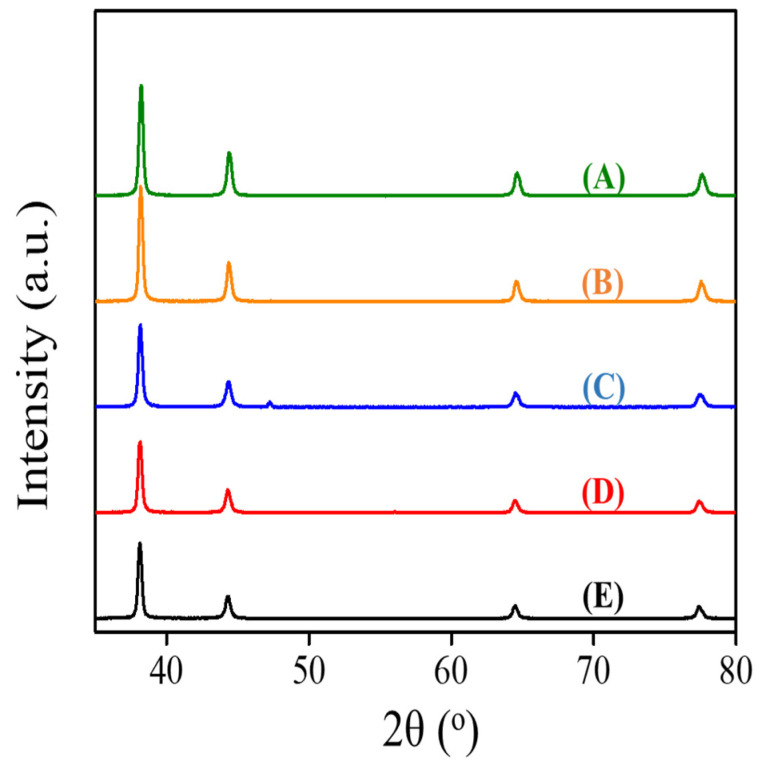
XRD data of various Au@Ag-DNFs/Si samples.

The XRD data of various Au@Ag-DNFs/Si samples are presented in Figure A1 Both Au and Ag have face-centered cubic (FCC) crystal structures with cell dimensions of 4.07 Å and 4.08 Å, respectively [45]. Therefore, the synthesized nanomaterials of Au, Ag and their composited nanomaterials can have similar XRD peaks [46]. The XRD data indicated that Au and Ag could be synthesized in a cocrystal structure and have high mechanical stability.

**Figure 6:**
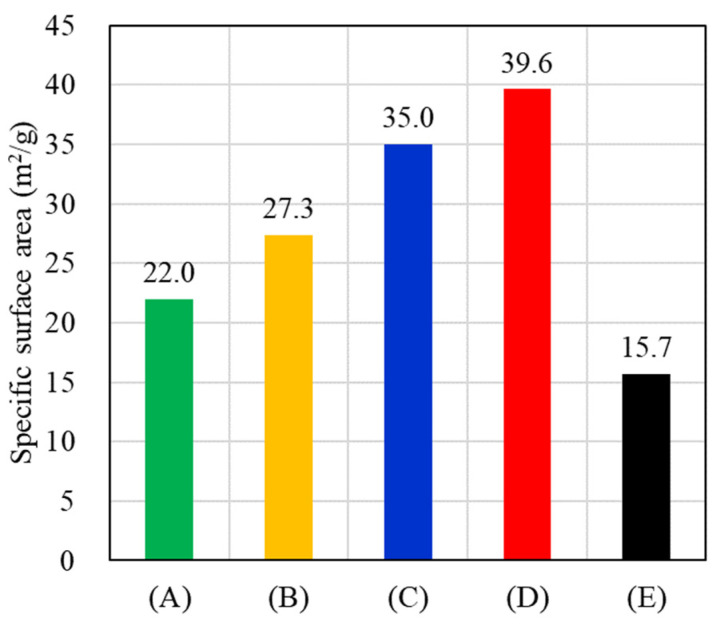
Specific surface area of various samples (A–E) in Figure 3.

As shown in Figure 6, BET analysis is assumed to be sensitive to multilayer adsorption on the external surface area [43]. The BET measurements presented that the increase of Ag-shell on Au-core resulted in increased specific surface area for samples (A–D). However, the synthesized pure Ag DNFs, sample (E), have the smallest specific surface area.

**Figure 7:**
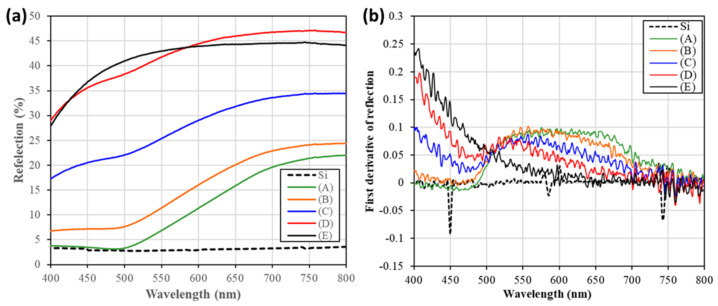
The (**a**) reflection and (**b**) first derivative spectra of Au@Ag–DNFs/Si samples (A–E) and the Si substrate.

The visible light (400–800 nm) reflection spectrum indicated the optical responses of various Au@Ag-DNFs/Si samples, as illustrated in Figure 7a. The reflection spectra collected the light of multiple wavelengths after direct reflection and the leakage light after multiple scattering inside an optical integrator. The optical reflection of different Au@Ag-DNFs/Si samples decreased with increased amounts of deposited Ag for all Ag-DNFs/Si samples except for sample (E). The edge in reflection spectrum data blue-shifted as the Ag deposition ratio increased.

Furthermore, the optical reflection spectra revealed the plasmonic response of the light-illuminated material. The optical plasma wavelength of Ag and Au are 400 nm and 520 nm, respectively, and the plasma wavelength of Ag–Au composited material can be tuned using different component ratios [47,48]. The first derivative spectra, see Figure 7b, of Au@Ag-DNFs/Si samples (A–E) and the Si substrate presented that the addition of Ag ratio resulted in a blue-shift of the plasmonic resonance peak. This means that the dominant near-field plasmonic response, excluding the light trapping effect by DNFs, transfer to peaking at around 532 nm, the wavelength of triggering light of SERS measurements.

DNF structures of dense leaves and branches resulted in the optical reflection of incident light. Illuminating light penetrates the gaps between the sharp leaves of the Au core and becomes trapped through the multiple scattering in the deep forest. The trapped light can convert to heat with plasmon-phonon interaction or the energy for enhancing chemical reaction or optical sensing. However, small Ag NPs can function as nanoantennas of high-scattering cross-sections to increase the reflection of light. According to the SEM data in Figure 3, Au-rich samples exhibited sharp leaves and branches, whereas the Ag-rich samples exhibited round leaves aggregated on the metal branches. The Ag round leaves exhibited higher reflectivity than did Au sharp leaves.

**Figure 8:**
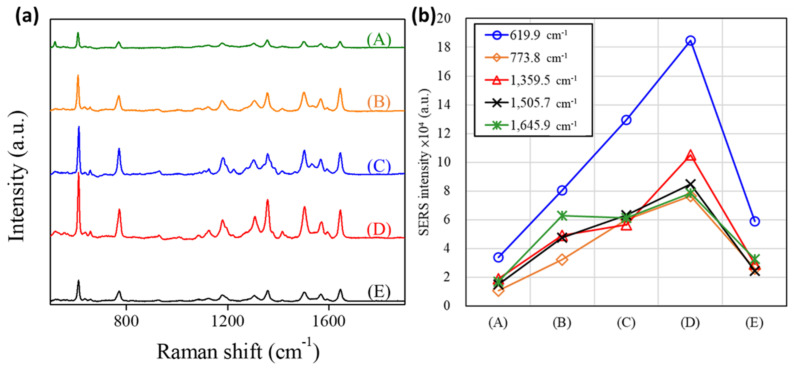
(**a**) Raman spectra of aqueous solutions of R6G molecules (10^−6^ M) on various Au@Ag−DNFs/Si substrates with the following ratios: (A) 250/0, (B) 190/60, (C) 125/125, (D) 60/190, and (E) 0/250 with an acquisition time of 0.5 s each. (**b**) R6G SERS intensity peaks for various samples. The wavelength of incident light is 532 nm with a power of 0.1 mW focusing in a 700 nm spot-width while Raman scattering signal accumulation time of 0.5 s.

SERS spectra of R6G molecules (10^−6^ M) in an aqueous solution were deposited on various Au@Ag-DNFs/Si substrates Figure 8a. The prominent peaks of R6G SERS responses in this work were 612.7, 772.1, 1184.0, 1304.7, 1359.5, 1502.5, 1569.9, and 1645.9 cm^−1^ and were identified using the results of samples (A–E). The increase in the Ag shell led to an increased SERS response in various samples (A–D) with Au core DNFs. However, the SERS response drastically decreased in the measurement with the sample (E) of the only Ag to construct DNFs. With a 60/190 Au/Ag ratio, sample (D) exhibited the most significant SERS response (see Figure 8b), which was 184,708 for the peak at 612.7 cm^−1^; this response exceeded that of samples (A) and (E), which were of pure Au and Ag, respectively. Therefore, Au core DNFs can enhance the SERS response of the Ag shell.

**Figure 9:**
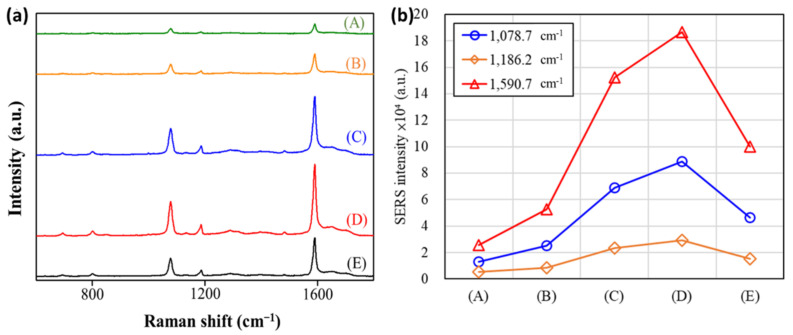
(**a**) Raman spectra of aqueous solutions of 4-MBA molecules (10^−6^ M) on various Au@Ag-DNFs/Si substrates with the following ratios: (A) 250/0, (B) 190/60, (C) 125/125, (D) 60/190, and (E) 0/250 with an acquisition time of 0.5 s each. (**b**) 4-MBA SERS intensity peaks for various samples. The wavelength of incident light is 532 nm with a power of 0.1 mW focusing in a 700 nm spot-width while Raman scattering signal accumulation time of 0.5 s.

Similar results were also found when using 4-MBA molecules, as Raman report. Figure 9a represents the SERS spectra of 4-MBA on samples (A–E). The two strong bands at 1590 and 1074 cm^−1^ dominate the SERS spectra; they were assigned to *ν*_8a_ aromatic ring vibrations and *ν*_12_ aromatic ring vibrations with C–S stretching characteristics, respectively. Sample (D) with a 60/190 Au/Ag ratio exhibited the most significant SERS response (see Figure 8b), which was 186,375 for the peak at 1590 cm^−1^.

Additionally, Raman EF value was found using the following equation:

(2) EF =ISERSNSERSINORNNOR

where *I_SERS_* and *I_NOR_* are the Raman intensities of SERS and normal Raman spectrum of the same Raman peak for R6G and 4-MBA molecule, respectively. *N_SERS_* and *N_NOR_* represent the corresponding number of R6G and 4-MBA molecules exposed to the laser-focused spot area. Because the laser parameters adopted in the SERS measurement were the same, *N_SERS_* and *N_NOR_* can be approximately determined by the concentration of R6G and 4-MBA, and this empirical calculation has been verified in many previous works. Based on a more accurate calculation [41] as shown in Appendix B, the values of *N_SERS_/N_NOR_* for R6G and 4-MBA by 532 nm laser used are found in Appendix C. According to this equation, the EFs of SERS for R6G and 4-MBA molecules were estimated as shown in Table 1, indicating that sample (D) had the highest EFs (R6G: 3.62 × 10^6^, 4-MBA: 1.76 × 10^7^) compared to other samples, and can be regarded as an SERS active substrate.

## 4. Discussion

SERS could be processed with the following steps: (i) electromagnetic wave focusing, (ii) light-material interaction, and (iii) electromagnetic wave retrieving. Step (i) is light focusing on nano features and induces various types of plasmons. Step (ii) is related to the polarizability of the surface-molecule complex as chemically or physically accepting the energy from the illumination light. Step (iii) is associated with the inelastic decay of generated plasmons, or focused light of hot spots of localized oscillating electrons, delivering or acquiring energy from the target material, e.g., R6G in this work, in Raman scattering measurements [49]. Steps (i) and (iii) are related to the plasmonic property of the SERS sensor (e.g., rough metal surfaces, metal NPs, periodical metal structures, or metal networks of metal nanowires or nano branches). Step (ii) is related to the quantum property of the target molecules and the SERS sensor of nanomaterials. Therefore, the SERS enhancement difference of various Au-to-Ag ratio DNFs samples can be discussed based on the above three SERS process steps.

De Barros et al. [42] presented the theoretical evaluation of the SERS enhancement factor, $F\left(\lambda \right)$, by boundary element method (BEM) numerical calculation. The SERS enhancement factor, related to numerous wavelengths of light, can be evaluated using near-field electromagnetic field intensity; see Equation (1) of [42]. ${\left(\frac{E_{loc}}{E_{0}}\right)}_{\lambda_{0}}^{2}$ represents the focusing efficiency of the source light in step (i). ${\left(\frac{E_{loc}}{E_{0}}\right)}_{\lambda }^{2}$ represents the retrieving efficiency of the SERS light in step (iii). The increased local electromagnetic field $E_{loc}$ of source light and SERS light resulted in increased SERS enhancement factor. In a finite-difference time-domain (FDTD) simulations, Chan et al. [17] presented that the external illuminating light can be focused into the tiny areas of the ends or in gaps of the silver nano branches. The high SERS response for sample (A–D) lead to a possibility of increased Ag coverage of Au@Ag-DNFs/Si samples can increase $E_{loc}$ in SERS measurement.

Furthermore, the reflection spectra data, graphed in Figure 7, provides more information on Ag coverage on Au can enhance SERS efficiency. The reflection spectrum roughly reflects the combined effects of the step (i) and (iii) for various wavelengths of light “in this work.” The multiple focusing and defocusing process of the propagating light inside the thick Au@Ag-DNFs layer in summation leads to incident light reflection. The increased ratio of Ag presented lower energy loss in the multiple reflection process. The increased Ag coverage thickness also led to higher reflection and higher chances to induce Raman signal in step (ii). This is consistent with the trend of SERS measurement results of sample (A–D).

Step (ii) of plasmon-material interaction occurs through both short-range chemical enhancement and physically through long-range electromagnetic enhancement of metal nanomaterials [4,6]. Long-range electromagnetic enhancement is caused by LSPR under light excitation on or near the surface of metallic NPs, thereby enhancing the local electromagnetic field [6,50]. Thus, the metal nanostructure and SERS material components play essential roles in generating strong SERS signals [6,50]. The elastic decay of plasmons causes scattering light and enhances step (iii) in SERS. However, the excessively dense round spheres in the Ag DNF layer can also improve the trapping of generated SERS light, as all Au DNFs can, in step (iii), which quenches the final scattering light propagated to the far-field and acquired by the detector. However, the sample (E) of pure Ag presented compatible high reflection but remarkably decreased SERS response compared with sample (D). The high reflection of sample (E) means low quenching of light in steps (i) and (iii). The intensity of localized surface plasmons or focused hot spots of light is typically stronger on Ag than on Au under external light illumination. Under resemble quenching effect, the use of light can be similar on Ag and Au@Ag DNFs. Therefore, the above discussions relating to step (i), (iii), and long-range enhancement in step (ii) cannot explain why the SERS response of Ag is smaller than Au through the intensity difference of localized electromagnetic field in this research.

As shown in Figure 6, the specific surface area of the sample (D), 39.6 m^2^/g, is 2.5 times larger than that of the sample (E), 15.7 m^2^/g. The chemical enhancement in step (ii) relies on conducting the light-generated high energy hot electrons on the metal surface to the target molecules. Among the metals used, Ag has been demonstrated to have a more significant EF than Au due to the absence of interband absorption [36,37,38,39]. However, the target molecules need to contact the surface of the SERS sensors. The eliminated specific surface area of the sample (E) decreased the number of fixed target molecules compared with sample (D). This makes sample (E) lower SERS signal generation efficiency in both the chemical enhancement and electromagnetic enhancement models in step (ii), even though pure Ag has a superior plasmonic response.

Therefore, sample (D) had the highest SERS response is the combined effect of both the electromagnetic enhancement and chemical enhancement. The highest enhancement factors by sample (D) reached 3.62 × 10^6^ and 1.76 × 10^7^ for analytes R6G and 4-MBA, respectively. The SERS enhancement results presented that the Au@Ag-DNFs showed a more robust SERS response than pure Au- or Ag-DNFs and are consistent with previous works [1,3,6,35]. However, the mechanism for the Au@Ag-DNFs of much larger feature sizes up to μm can be different from that of nanometer-sized Ag–Au composited nanoparticles.

## 5. Conclusions

High-density Au@Ag-DNFs on a Si substrate was synthesized using the FAGRR method. Au@Ag-DNFs/Si with various Au/Ag ratios of samples (A) 250/0, (B) 190/60, (C) 125/125, (D) 60/190, and (E) 0/250 were synthesized to evaluate their SERS response. SEM results demonstrated an increase in round-shaped nanostructures with the additional Ag coverage. The UV-visible reflection spectrum revealed increased reflection and light induction in response to the addition of Ag when creating the metal DNF layer. The pure Ag DNFs strongly reflected visible light but exhibited low SERS response. A Au core in Au@Ag DNFs enhanced the high plasmonic response of the Ag coverage. The pure Ag DNFs of spherical leaves lacked a Au core, and hence, could not maintain the long leaves and thereby focus illumination light further. Therefore, the SERS response of Ag DNFs was higher than that of Au DNFs but was lower than that of Au@Ag DNFs.

## Figures and Tables

**Table 1:** SERS enhancement factors of the samples (A–E).

Name	*I_NOR_*	*I_SERS_*	EFs
R6G	186	-	-
Sample A	-	42,652	8.37 × 10^5^
Sample B	-	101,932	2.00 × 10^6^
Sample C	-	135,499	2.66 × 10^6^
Sample D	-	184,708	3.62 × 10^6^
Sample E	-	58,685	1.15 × 10^6^
4-MBA	142	-	-
Sample A	-	25,557	2.41 × 10^6^
Sample B	-	52,668	4.97 × 10^6^
Sample C	-	153,544	1.45 × 10^7^
Sample D	-	186,475	1.76 × 10^7^
Sample E	-	100,107	9.45 × 10^6^

## Appendix B

Calculation of SERS enhancement factors [41].

λ = wavelength of the laser used	532 nm	
NA = numerical aperture	0.90	
ρ = density of analyte molecule	R6G: 1.26 g/cm^3^	4-MBA: 1.49 g/cm^3^
Molecular weight	R6G: 479 g/mole	4-MBA: 154 g/mole
Area of the sample	9 cm^2^	
Laser spot diameter ω0=1.22λNA	7.21 × 10^−5^ cm	
Laser focal depth z0=2πλω02	6.14 × 10^−4^ cm	
Laser focal volume τ=π232ω02z0	6.3× 10^−12^ cm^3^	

(A1) NNOR=confocal volume×densitymolecular weight×Avogadro′s number NA

(A2) NSERS=laser spot areasubstrate area×volume V×concentration C×Avogadro′s number NA

## Appendix C

*N_NOR_*, *N_SERS_*, and ratio of *N_NOR_* to *N_SERS_* for R6G and 4-MBA.

**Raman Molecule**	**R6G**	**4-MBA**
*N_NOR_*	9.98 × 10^9^	3.67 × 10^10^
*N_SERS_*	2.73 × 10^6^	
*N_NOR_/N_SERS_*	3.65 × 10^3^	1.34 × 10^4^
